# Women after Bilateral Surgical Correction of Hallux Valgus Do not Show Improvement in Spatiotemporal Gait Parameters at 18 Weeks Postoperatively

**DOI:** 10.3390/jcm10040608

**Published:** 2021-02-05

**Authors:** Katarzyna Kaczmarczyk, Gabor J. Barton, Ida Wiszomirska, Michal Wychowanski

**Affiliations:** 1 Faculty of Rehabilitation, Józef Piłsudski University of Physical Education in Warsaw, 00-968 Warsaw, Poland; ida.wiszomirska@awf.edu.pl (I.W.); michal.wychowanski@awf.edu.pl (M.W.); 2 Research Institute for Sport and Exercise Sciences, Liverpool John Moores University, Liverpool L3 3AF, UK; G.J.Barton@ljmu.ac.uk

**Keywords:** gait analysis, spatiotemporal parameters, hallux valgus

## Abstract

Background: Hallux valgus (HV) is a gait-altering orthopedic deformity, somewhat more prevalent in women, which often affects both limbs. Although surgery is a commonly applied treatment, there is no consensus in the literature on how invasive HV correction affects spatiotemporal gait parameters, or how quickly improvement can be expected. We investigated gait parameters in female HV patients who underwent bilateral surgical correction of hallux valgus, both preoperatively and 18 weeks following surgery (a timeframe relevant from the perspective of physical therapy), and also in relation to a non-HV control group. Methods: A total of 23 women aged 40–70 years, with moderate to severe HV deformity in both feet, were assessed preoperatively and 18 weeks postoperatively, and an age-matched control group of 76 healthy women was also assessed. A total of 22 spatiotemporal parameters were collected during 30 s walks over an electronic walkway (Zebris Medical System). Results: Of the 22 parameters analyzed, significant differences between the preoperative experimental and control groups were found only in 4 parameters (Velocity, Right step time, Total double support and Stride time), but in 16 parameters between the postoperative experimental and control groups (the greatest impact being found for: Left and Right Step time, Stride time, Cadence, Right Foot rotation, Left Step length (% leg length) and Stride length (% leg length)). Conclusions: Women after bilateral HV correction did not exhibit improved (i.e., more normal) gait parameters at 18 weeks postoperatively; rather, they showed more gait abnormalities than preoperatively. These findings urge longer-term planning of postoperative rehabilitation, involving continual evaluation of gait improvement.

## 1. Introduction

Hallux valgus (HV) is a common orthopedic deformity encountered in clinical practice that affects 23% to 38% of the population in general, with a higher prevalence in women (30%) than in men (13%) [1]. HV is recognized as a major public health problem and as a source of notable problems in women, such as osteoarthritis, greater risk of falling, lesser quality of daily life, etc. [2]. The deformity is characterized by the subluxation and valgus angulation of the first metatarsophalangeal joint (1MTPJ) in combination with the pronation of the proximal phalanx. Deschamps et al. [3] have reported that genetic predisposition, wearing inappropriate shoes, trauma and biomechanical compensation for structural and functional deformities may contribute to this disorder; Pérez Boal et al. [4] have proposed that HV development involves a skeletal parameter of the first metatarsal bone and proximal phalanx hallux. HV is progressive in nature, and at the advanced stage, it is known to impact kinematic and kinetic parameters of gait due to continual pain and discomfort [5]. Several authors have demonstrated gait deviation in HV patients [3,5,6,7]; however, studies report inconsistent findings. 

Treatments vary: There are a wide variety of conservative treatment options [8,9], but surgical intervention is quite common, with more than 100 different invasive correction techniques having been described [10,11,12]. Moreover, HV is reported to be bilateral in 84% of cases [13]; the majority of patients require surgical correction on both feet, and some researchers have provided evidence in favor of simultaneous surgical correction [14,15].

The effectiveness of the specific surgical procedures has, in most cases, been evaluated by questionnaires and radiological examination [16,17,18], whereas only a limited amount of information exists regarding the influence of HV correction surgical procedures on gait parameters [7,19,20,21,22,23]. Authors have found higher functional scores and less pain demonstrated in most patients; however, there is no agreement as to whether hallux valgus correction actually improves spatiotemporal gait parameters. Stevens et al. [23] reported no significant differences in gait velocity, stance time or step length between an HV group after surgery and a control group. The results of Canseco et al. [20], Kuni et al. [22] and Klugarova et al. [7], in turn, indicate that HV operation negatively affects spatiotemporal parameters. In contrast, Moerenhout et al. [19] and Brodsky et al. [21] reported improvement in spatiotemporal outcomes and restoration to a normal gait pattern postoperatively.

Note that, while most of these studies were carried out at least a year after surgical intervention, rehabilitative procedures typically assume progress in gait recovery over much shorter durations, for instance resuming normally weighted gait at 15 weeks. Moreover, although HV is more prevalent in women, no report in the literature specifically offers evidence as to whether gait parameters improve in women treated for bilateral, moderate to severe HV deformity, within a relatively short timeframe postoperatively—information of potentially great significance for rehabilitative practice and scheduling.

The aim of this study, therefore, was to investigate spatiotemporal gait parameters in women with bilateral, moderate to severe HV deformity, comparing them preoperatively vs. at 18 weeks following surgery and comparing each of these in relation to a control group. We sought evidence for whether spatiotemporal gait parameters for the experimental group actually improved (understood as becoming more normal, i.e., like those of the control group) within this therapeutically relevant timeframe.

## 2. Materials and Methods

This study (with a nonequivalent pretest-posttest control group design) was carried out at the Central University Laboratory, the University of Physical Education in Warsaw, Poland. Recruitment of patients into experimental and control groups was carried out based on availability (limitation section).

### 2.1. Participants

Twenty-three females with HV deformity in both feet (classification according to the American Orthopaedic Foot and Ankle Society) [24] met the inclusion criteria: age 40–70, moderate to severe HV deformity, without any other lower limb disease. The exclusion criteria were any other lower limb pathologies (e.g., muscle weakness, foot-drop, ischemic disease), previous surgeries (e.g., surgical fixation of foot or ankle fractures) or pain, which could all affect their gait, as ascertained by a medical interview. For the control group, 76 healthy women qualified, corresponding in age to the experimental group.

All subjects were informed about the purpose of the study and provided written informed consent. Approval was obtained from the Ethics Committee of the Józef Pilsudski University of Physical Education in Warsaw (SKE 01-33/2019), in accordance with the guidelines specified in the Declaration of Helsinki on human experimentation.

### 2.2. Procedures

In the experimental group, all subjects were assessed twice. The preoperative examination (pretest) involved clinical orthopedic, X-ray and MRI and anthropometric examination (body height, weight and lower limb length measured from the greater trochanter of the femur to the lateral malleolus) and gait assessment. The postoperative examination (posttest) consisted of a gait assessment.

The patients in the experimental group then underwent first metatarsal osteotomy by four different methods (Chevron, Scarf, Oblique, Semicircular), all of them having both limbs operated on at the same time. The operation was followed by therapy overseen by a physical therapist, in accordance with the following physiotherapeutic procedure. For the first four weeks, it involved rest, the use of orthopedic shoes, walking and anticlotting exercises. The next stage involved the mobilization of healed fragments of scar tissue and first MTP extension, therapy of the soft tissues of the underside of the foot, mobilization of the sesamoid bones and exercises strengthening the flexors and extensors of the knee joint. In the sixth week, weight-bearing and gait re-education began, together with exercises strengthening the peroneus longus muscle and sitting calf raises. In the eighth week, therapy involved exercises strengthening the flexors and extensors of the hallux with resistance, dynamic exercises. In the tenth week, it involved calf raises, with running starting around the fifteenth week.

Kristen et al. [25] state the time from surgery to return to work is 5.8 weeks and to sport 8.4 weeks. In our postoperative physiotherapeutic procedure, full weight-bearing may occur after 6 weeks and return to sport after 15 weeks. As such, the postoperative gait evaluation in our study took place with a mean of 18 weeks postoperatively, after complete return to a stable ambulatory pattern.

**Figure 1:**
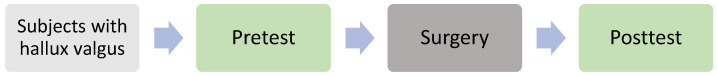
Study flow chart for the experimental group.

The procedure with the experimental group took place according to the following flow chart (Figure 1).

In the control group, in turn, a single examination involved an assessment of anthropometric parameters (body height, weight and lower limb length as mentioned above) and gait assessment.

### 2.3. Gait Assessment

Gait performance was measured on a 304 cm long, 56 cm wide electronic walkway (Zebris Medical System, Tübingen, Germany) [26]. Data were sampled at 120 Hz and stored in a personal computer, which calculated spatiotemporal parameters and foot pressure distribution parameters using the Zebris software. All data collection was conducted at the Central University Laboratory. After 2 practice trials for familiarization, the individuals performed 3 walking trials (to eliminate the wayward effect during initiation and termination of walking), at their normal velocity. Twenty-two spatiotemporal parameters were collected over a 30 s capture period, equating to an average of 52 ± 5 steps of steady-state walking. These spatiotemporal parameters were as follows: Left and Right step length (% of leg length); Left and Right foot rotation (degrees); Stride length (% leg length); Step width (cm), Left and Right step time (s); Left and Right stance phase (% of gait cycle (GC)); Left and Right loading response (%GC); Left and Right single support (%GC); Left and Right pre-swing (%GC); Left and Right swing phase (%GC); Total double support (%GC); Stride time (s); Cadence (strides/min); Velocity (km/h). The foot pressure distribution parameters were: Gait line length left (mm), Gait line length fight (mm), Single support line left (mm), Single support line right (mm), Ant/post position (mm), Lateral symmetry (mm).

The patients performed the walking trials barefoot and unaided, each time starting and finishing walking 2 m before and after the mat to minimize acceleration and deceleration effects.

### 2.4. Statistical Analysis

Data (analyzed with STATISTICA version 13, PL.iso) are expressed as mean ± standard deviation. The Shapiro–Wilk test showed that most of the studied parameters did not exhibit a normal distribution. To compare the results of the experimental group between the first and second test (preoperatively and postoperatively), the non-parametric Wilcoxon test was applied. To compare the experimental and control groups, the Mann—Whitney U test was applied. To avoid statistical bias, the Bonferroni correction was applied.

Statistical significance was set at *p* < 0.05. Multi-feature profiles were also made, i.e., graphical representation of average values of gait parameters for the study groups, which allowed for an effective visual assessment of their mutual configuration rather than a review of the same data included in the table. The calculated gait parameters had different scales and distributions; hence, to make them comparable, z-scores were calculated (experimental data—mean of control data/standard deviation of control data) for each gait parameter.

The estimated number of participants was calculated taking into account: α = 0.05, target power 0.8, effect size 0.8 and number of groups 2. The number of participants (sample size) in the experimental group for pre- vs. postoperative comparisons (dependent variables), with pre-/post correlation r = 0.4, was 17. The number of participants in the single group for experimental vs. control comparisons (independent variables) was 26. Estimation of the number of participants was carried out using Statistica v. 13 (TIBCO Software Inc., Palo Alto, CA, USA) for the family of *t*-tests.

## 3. Results

Demographics of the study population at baseline are presented in Table 1.

There were no significant differences with respect to the age, body mass, height and BMI between the experimental and the control groups. HVA (angle between long axis of 1st metatarsal and the longitudinal axis of the first proximal phalanx) values were 26–48 min–max, IMA (angle between long axis of 1st and 2nd metatarsal) values 12–17 min–max.

### 3.1. Spatiotemporal Parameters before Surgery (Preoperative HV vs. Control Group)

First, we compared the parameters of the preoperative experimental vs. control group. Out of 22 parameters analyzed, significant differences between these groups were found only in terms of 4 parameters: Velocity (Z = −2.20; *p* = 0.026, d = 0.46), Total double support (Z = 1.98; *p* = 0.046, d = 0.47), Right Step time (Z = 2.50; *p* = 0.012, d = 0.67) and Stride time (Z = 1.99; *p* = 0.045, d = 0.44). Patients with HV deformity were found to walk more slowly, with lower cadence, reduced duration of stride time and reduced right step time as compared to the control group (Table 2).

### 3.2. Spatiotemporal Parameters after Surgery (Postoperative HV vs. Control Group)

Comparative analysis of the HV group after surgery to the control group, in turn, revealed significant differences in terms of more parameters (Table 2). To see which parameters were affected most, the effect size was calculated (Cohen’s d). Compared to the control group, the intervention had the greatest impact on: Velocity (Z = −5.34; *p* < 0.000, d = 1.4), Left (Z = 4.54, *p* < 0.000, d = 1.25) and Right step time (Z = 4.67; *p* < 0.000, d = 1.25), Stride time (Z = 4.53; *p* < 0.000, d = 1.24), Cadence (Z = −4.46; *p* = 0.001, d = 1.21), Right foot rotation (Z = 4.53; *p* = 0.000, d = 1.14), Left step length [%leg length] (Z = −4.69; *p* < 0.000, d = 1.1) and Stride length [%leg length] (Z = −4.40; *p* = 0.0001, d = 1.05).

### 3.3. Spatiotemporal Parameters before and after Surgery (Preoperative vs. Postoperative HV Group)

**Figure 2:**
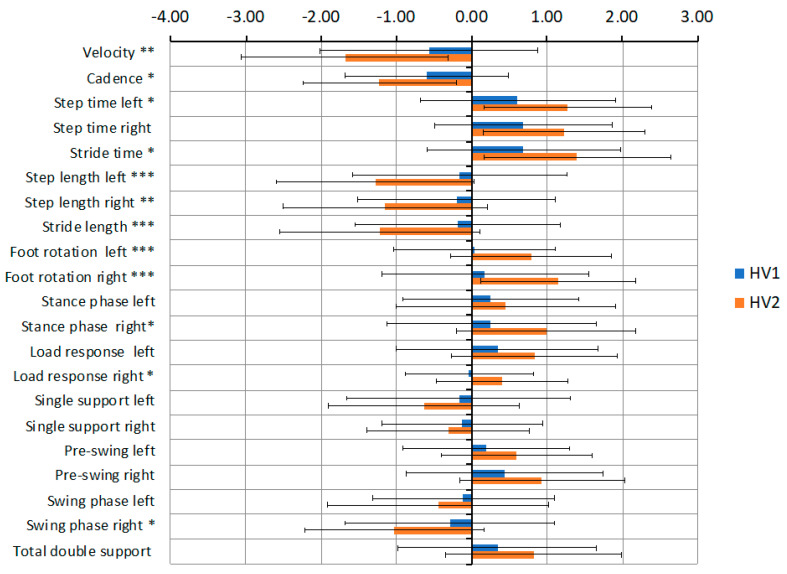
Differences in gait parameters of z-scores in the patient group preoperatively (HV1) and postoperatively (HV2). * *p* < 0.05, ** *p* < 0.01, *** *p* < 0.001.

In the next step, the differences between spatiotemporal parameters in the HV group before and after surgery were assessed. To illustrate the differences in gait parameters before and after surgery, a profile showing the change of the standardized z-scores was created (Figure 2). Significant changes were observed in Velocity (*p* < 0.01), Cadence (*p* < 0.05), Left step time (*p* < 0.05), Stride time (*p* < 0.05), Left step length (*p* < 0.001), Right step length (*p* < 0.01), Stride length (*p* < 0.001), Left foot rotation (*p* < 0.001), Right foot rotation (*p* < 0.001), Right stance phase (*p* < 0.05), Right loading response (*p* < 0.05) and Right swing phase (*p* < 0.05).

In the experimental group, the pressure distribution along the sole of the foot during each step was measured. In most parameters, significant changes were observed. The greatest differences in HV patients were demonstrated in the left (*p* < 0.001) and right (*p* < 0.01) single support line and the sagittal plane movement of the CoP (CoP-centre of pressure) (*p* < 0.01); Table 3.

### 3.4. Impact of Surgery Method on Gait Parameters in HV

Finally, in the last stage of analysis the impact of the particular surgical procedure on the postoperative results was checked (as a pilot study). In the experimental group, subjects with HV had been operated on using four different methods: Chevron, Scarf, Oblique and Semicircular. We selected for analysis the two most frequently represented interventions: Oblique (*n* = 10) and Chevron (*n* = 7). Statistical analysis showed no differences between the groups in all parameters analyzed before surgery. In the second study (after surgery) significant differences at *p* < 0.05 were found only in terms of two variables: Left Pre-swing (%GC) and Left Loading response (%GC), which showed lower values in the group of patients operated on using the Chevron intervention.

## 4. Discussion

The aim of our study was to investigate spatiotemporal gait parameters in women with bilateral, moderate to severe hallux valgus deformity, comparing them preoperatively vs. 18 weeks postoperatively and comparing each of these in relation to a control group. The main objective was to test if bilateral surgical treatment of HV patients improves their gait pattern within the short postoperative term of 18 weeks, a timeframe relevant from the perspective of the planning and practice of physical therapy. Overall, we found that HV surgery had a negative effect on gait parameters within this short postoperative horizon. Significant changes were found in most spatiotemporal parameters compared to the control group. Patients who underwent corrective osteotomy for HV exhibited reduced Velocity, Cadence, Step length, Stride length, Swing and Single support as well as lengthened Step time, Stride time, Stance phase, Loading response, Pre-swing, Foot rotation and Double support.

Such a lack of improvement in gait parameters (understood as abnormality in more, rather than fewer parameters) in the short term after surgical correction could, we conjecture, be due to pain, apprehension against loading the limb, fixed altered gait patterns and the examinations being carried out too soon after the procedure. Similar results have been reported in other studies [7,20,22,27]—although they noted clinical and radiological improvement, this did not appear to go hand-in-hand with restoration of normal gait.

Gait deviation in patients with hallux valgus, including spatiotemporal parameters, has been documented in the literature; however, the systematic review by Nix et al. [28] indicates that results do vary. Some authors have investigated these parameters and demonstrated no significant differences between HV patients and a control group [3,29], while other findings, in contrast, showed significant differences in walking speed, step length [6], cadence, foot-flat, push-off, double support, speed [5], velocity, step length and stride length [7], velocity, stride length and stance %GC [20] in an HV group as compared with a control group. Our results show that hallux valgus does indeed affect spatiotemporal variables. Among the preoperative spatiotemporal results of the current study, five parameters, those related to the temporal nature of gait, demonstrated significantly lower values when compared to controls. These findings are consistent with previous studies [5,6,7,20]. According to Menz and Lord [6], these differences in spatiotemporal variables due to HV affect gait patterns may cause instability and risk of falling.

Surgical interventions alter the biomechanics of the foot and the function of the first ray [30]. The resulting stiffness of the metatarsophalangeal joint and consequently the medial arch of the foot disturbs the weight transfer and causes reduced power generation in the push-off phase. These adverse changes may be due to the pain that is still present in the operated foot. In short-term postoperative patients (ten weeks postoperative), Sadra et al. [27] found a significant reduction in walking velocity compared to the pre-operative speed and control group. At four months postoperatively, Klugarova et al. [7] showed a significant decrease in step time and walking speed in the operated leg, and even more changes were observed in the non-operated leg after HV surgery, probably due to pain and apprehension against loading the operated leg. Menz et al. [6] revealed that foot pain is significantly associated with difficulty performing various weight-bearing activities. Similar conclusions were drawn by Moerenhout et al. [19], who reported that longer contact time on the lateral border of the foot and at the medial forefoot to dampen the push-off and prevent hallux loading was observed in HV patients in the short term after surgery (within 6 months). We also observed this in our research, in which the single support line for the right and left limb was significantly shorter at 18 weeks postoperatively as compared to preoperatively, which attests to a flat positioning of the foot during first contact and restricted propulsion.

It would appear that the timing of postsurgical examination has a great influence on the results. Moerenhout et al. [19] showed that gait parameters continue to improve at 12 months postoperatively, and this improvement is expected to continue after 12 months. Maximal force and pressure at the second and first toe were seen to improve at 12 months compared to the six-month follow-up. This suggests that patients started walking more confidently and putting more weight on the operated joint. Similar results were presented by Brodsky et al. [21], who demonstrated three significant changes in gait in HV patients at least 12 months after operation: increases in maximal ankle push-off power and single-limb support time on the involved lower limb, and a decrease in step width. These changes improve propulsive power, weight-bearing function of the foot and stability during gait.

Most of the above studies were longer-term, carried out at least a year after surgical intervention. This may explain the lack of improvement seen in our results, recorded just 18 weeks post-surgery. The findings of Moerenhout et al. [19] and Brodsky et al. [21] suggest long-term postoperative rehabilitation following HV intervention. However, Saro et al. [31] showed that values of spatiotemporal parameters in the long-term postoperative period may depend on the surgical method used—they reported that the walking speed in patients operated by the Lindgren method is much lower than those who received a Chevron osteotomy. King et al. [32], in turn, reported differences in forefoot distribution in patients operated on by either the Lapidus or Chevron procedures. In our study, we only compared patients who had undergone the Chevron and Oblique methods and found significant differences in only two gait parameters postoperatively; the reason for this likely lies in the small number of subjects in each group.

As for the limitations of this study, one that should be noted is that recruitment of patients into experimental and control groups was carried out based on availability, and the groups differ in numbers. However, there were no significant differences with respect to the age, body mass, height and BMI between the experimental and the control groups. Other limitations include the lack of consideration of potential confounding factors, and the lack of registration in an international clinical trials register.

## 5. Conclusions

Evaluation of gait parameters is particularly important in individuals with HV, given that surgical treatment is opted for so commonly. However, there is no consensus among existing reports in the literature as to how surgery actually affects spatiotemporal gait parameters, or how quickly improvement should be expected. Moreover, no studies have reported on postsurgical gait specifically in female patients with bilateral, moderate to severe hallux valgus deformity.

We found that gait parameters in such patients did not return to normal levels at 18 weeks postoperatively (a therapeutically relevant timeframe); rather, they showed more gait abnormalities than preoperatively. Of the 22 parameters analyzed, significant differences were demonstrated only in 4 parameters between the preoperative experimental and control groups, but in 16 parameters between the postoperative experimental and control groups.

Overall, these findings suggest a need for longer-term planning of postoperative rehabilitation in patients undergoing bilateral surgical hallux valgus correction, involving more continual evaluation of gait improvement.

## Figures and Tables

**Table 1:** Demographics of the study population; results in mean ± SD.

Physical Characteristics	HV (*n* = 23)	Controls (*n* = 76)	*p*
Age (years)	55.49 ± 6.48	54.66 ± 6.83	0.604
Body mass (kg)	72.83 ± 10.40	68.59 ± 10.25	0.087
Body height (cm)	164.9 ± 4.33	163.9 ± 5.71	0.432
BMI (kg/m^2^)	26.71 ± 3.06	25.58 ± 3.93	0.207

HV—hallux valgus.

**Table 2:** Spatiotemporal parameters during the gait cycle in hallux valgus (HV) before and after and control groups.

N	Parameter	HV Before Surgery	HV After Surgery	Control Group
		Mean ± SD	Mean ± SD	Mean ± SD
1.	Velocity (km/h)	4.3 ± 0.69 ^#^	3.76 ± 0.66 ***	4.57 ± 0.48
2.	Cadence (strides/min)	57.2 ± 4.55	54.6 ± 4.24 ***	59.7 ± 4.18
3.	Step time left (s)	0.53 ± 0.05	0.56 ± 0.04 ***	0.51 ± 0.04
4.	Step time right (s)	0.54 ± 0.05 ^#^	0.56 ± 0.04 ***	0.51 ± 0.04
5.	Stride time (s)	1.06 ± 0.09 ^#^	1.11 ± 0.09 ***	1.01 ± 0.07
6.	Step length (%leg length), left	84.2 ± 9.72	76.6 ± 8.97 ***	85.3 ± 6.83
7.	Step length (%leg length), right	83.9 ± 9.18	77.3 ± 9.52 ***	85.3 ± 6.98
8.	Stride length (%leg length)	168 ± 18.8	154 ± 18.3 ***	171 ± 13.8
9.	Foot rotation (deg) left	4.78 ± 4.91	8.22 ± 4.88 **	4.62 ± 4.56
10.	Foot rotation (deg) right	6.10 ± 5.90	10.3 ± 4.40 ***	5.33 ± 4.30
11.	Stance phase (%GC), left	62.8 ± 2.35	63.5 ± 2.85	62.6 ± 1.95
12.	Stance phase (%GC), right	63.1 ± 2.29	64.3 ± 1.96 **	62.6 ± 1.65
13.	Load response (%GC), left	13.5 ± 2.45	14.4 ± 2.01 **	12.9 ± 1.83
14.	Load response (%GC), right	13.2 ± 1.88	14.2 ± 1.94	13.3 ± 2.22
15.	Single support (%GC), left	36.2 ± 2.53	35.4 ± 2.17 *	36.5 ± 1.71
16.	Single support (%GC), right	36.2 ± 2.27	35.8 ± 2.31	36.5 ± 2.14
17.	Pre-swing (%GC), left	13.7 ± 2.13	14.5 ± 1.95	13.3 ± 1.94
18.	Pre-swing (%GC), right	13.7 ± 2.31	14.6 ± 1.95 ***	12.9 ± 1.77
19.	Swing phase (%GC), left	37.2 ± 2.35	36.5 ± 2.85	37.4 ± 1.95
20.	Swing phase (%GC), right	36.9 ± 2.29	35.7 ± 1.96 ***	37.4 ± 1.65
21.	Total double support (%GC)	27.1 ± 4.18 ^#^	28.6 ± 3.71 **	26.0 ± 3.18
22.	Step width (cm)	7.83 ± 2.09	8.12 ± 1.84	7.22 ± 2.47

Legend: HV—experimental group with hallux valgus, CG—control groups, GC—gait cycle. ^#^ Differences between hallux valgus group before surgery and control group. * Differences between hallux valgus group after surgery and control group. * *p* < 0.05, ** *p* < 0.01, *** *p* < 0.001, ^#^ *p* < 0.05.

**Table 3:** Results of CoP cyclogram of the HV group before and after surgery.

N	Parameter	HV Before Surgery Mean ± SD	HV After Surgery Mean ± SD
1.	Gait line length left (mm)	214.30 ± 11.93	210.13 ± 15.06 *
2.	Gait line length fight (mm)	213.04 ± 14.13	215.07 ± 12.67
3.	Single support line left (mm)	134.61 ± 17.07	116.70 ± 22.61 ***
4.	Single support line right (mm)	133.48 ± 19.94	117.09 ± 23.12 **
5.	Ant/post position (mm)	121.83 ± 8.39	117.00 ± 9.27 **
6.	Lateral symmetry (mm)	−1.22 ± 6.19	−1.74 ± 5.01

* *p* < 0.05, ** *p* < 0.01, *** *p* < 0.001.

## Data Availability

The datasets during and/or analyzed during the current study are available from the corresponding author on reasonable request.
